# Research of System Design and Automatic Detection Method for Excretion Nursing Equipment

**DOI:** 10.3390/healthcare11030388

**Published:** 2023-01-29

**Authors:** Bingshan Hu, Zhiwei Chen, Xinyu Chen, Sheng Lu, Yingbing Su, Hongliu Yu

**Affiliations:** 1 Institute of Rehabilitation Engineering and Technology, University of Shanghai for Science and Technology, Shanghai 200093, China; 2 Shanghai Engineering Research Center of Assistive Devices, Shanghai 200093, China

**Keywords:** urination and defecation care, automation, multi-sensor fusion detection algorithm

## Abstract

(1) Background: The nursing of the elderly has received more and more attention, especially the nursing of urination and defecation for the elderly. (2) Purpose: Design an excretion nursing equipment that can accurately identify and deal with urine and stool. (3) Methods: In this paper, based on the analysis of the requirements of excretion nursing equipment, a split mechanical design method and a modular control method are used to design the equipment. The Dempster–Shafer (D-S) evidence theory is used in the identification of urine and stool. (4) Results: The excretion nursing equipment designed in this paper works well according to functional test, and the success rate of stool and urine identification method using D-S evidence theory is 20% higher than that of traditional methods, reaching 90%. (5) Conclusions: The urine and stool recognition and detection algorithm based on the D-S evidence theory used in this paper can improve the recognition accuracy of traditional detection methods, and the designed excretion nursing equipment can realize the function of excretion care for patients.

## 1. Introduction

**Figure 1:**
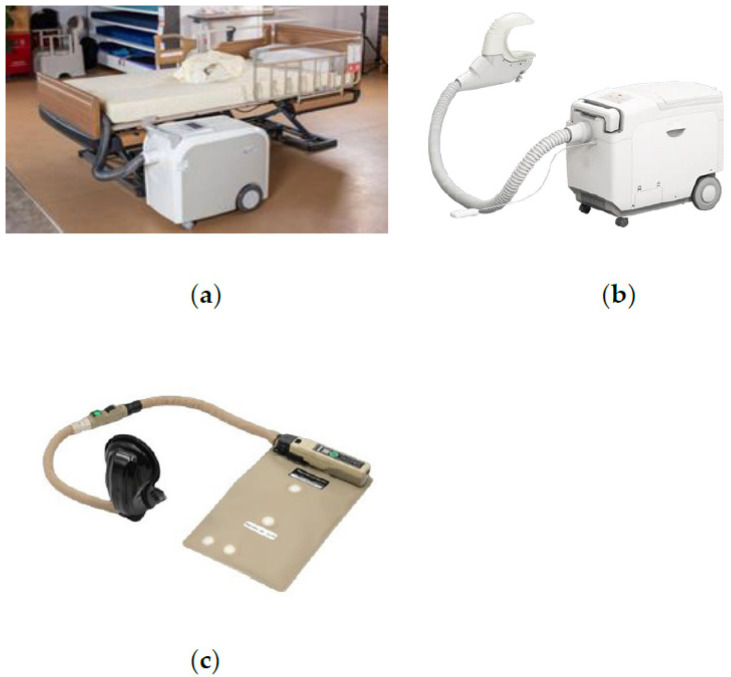
(**a**) Smilet automatic excretion processing robot; (**b**) Illinois Nursing Robot; (**c**) AMXDmax Urine collecting device [3].

The rapid development of population aging has attracted the attention of the United Nations and governments around the world. According to the survey, the global population aged 60 and above in 2017 was 962 million. By 2050, the number of people in this age group will be more than twice that of 2017, reaching 2.1 billion. By 2100, it will be more than three times that of 2017, reaching 3.1 billion. China is one of the countries with the fastest aging rate in the world. By the end of 2021, China’s elderly population aged 60 and above will reach 267 million, accounting for 18.9% of the total population. By 2035, the elderly population aged 60 and above will exceed 400 million, accounting for more than 30% of the total population, and will enter the stage of severe aging [1]. With the growing trend of population aging, the nursing of the elderly has received more and more attention. Due to the decline of physical function, the elderly is prone to various diseases, including incontinence and other maladies that make them difficult to care for. Therefore, intelligent excretion nursing equipment was developed. Compared with manual urination and defecation care, the intelligent excretion nursing equipment can detect and collect the user’s urine and stool in real time, and clean and dry the user’s excretion site, which can solve the problems of the dirty and working environment of nursing workers when nursing the defecation and urination of the elderly, awkward situations when the nurse’s and patient’s genders are different, and invasion of patient privacy. The more established urination and defecation care products on the market include the Smilet automatic excretion treatment robot (Figure 1a) developed in Japan and the China Suzhou Illinois Nursing Robot (Figure 1b). These two types of excretion nursing equipment, which are divided into two parts, i.e., a work head and a host, realize the functions of detecting the user’s urine and stool, flushing them, and washing and drying the human skin [2]. However, one problem of the current excretion nursing equipment is that the working head needs to be matched with a special mattress, and the versatility is insufficient. In addition, Omni Medical System has designed a pilot-specific urine collection device AMXDmax (Figure 1c). Macaulay M. et al. designed and developed a portable female excretion nursing device for convenient urine collection in order to solve the problems of difficult cleaning, heavy weight, and unreliability of traditional female excretion nursing equipment [3]. 

In addition to these products that have been on the market, scholars have also performed a significant amount of related research on urination and defecation care. Since most of the elderly users of urinary incontinence use disposable diapers to solve the problem of incontinence, and the amount of urine that the diapers can absorb is limited, when the diapers absorb enough urine, the problem of overflow and leakage will occur. To this end, B. Fernanders et al. designed an electronic system for urinary incontinence detection. The sensor is sewn into the underwear to detect the overflow of urine on the urine pad and send a signal to the wearer in time [4]. K. Nakajima et al. developed a urinary incontinence warning system for diapers. The entire system is installed outside the diaper, which avoids the cumbersome removal and installation of sensors during replacement. At the same time, it detects the wearer’s urinary incontinence and reminds the wearer when incontinence occurs [5]. A. Otero et al. designed an automatic urination recording device in order to help the hospital monitor the user’s urine volume and detect the user’s treatment effect, which avoids human errors during the operation and reduces the workload of nursing staff [6]. T. Fujimoto et al. developed a new automatic excretion detection system using reflected light sensor and impedance sensor, which can detect the excretion of the user automatically [7].

At present, most of the urinal nursing devices use the sensor to detect the urination and defecation directly. This method determines the type of urination and defecation according to the output data of the sensor directly, and the detection accuracy needs to be improved. The multi-sensor fusion algorithm can perform multi-level fusion processing on multiple different types of sensor data, so as to obtain more accurate detection results [8]. Since the detection object of the excretion nursing equipment is the urine and stool excreted by the user, the data fusion is carried out on the basis of the detection results of each sensor, so the decision layer fusion method is used. The existing decision-level fusion algorithm schemes include the Bayesian theory [9], Dempster–Shafer (D-S) evidence theory, expert system method [10], fuzzy theory [11], and so on. However, due to the real-time changing characteristics of the dynamic environment and the target, the difficulty in obtaining prior knowledge, etc., the application environment of expert system method and fuzzy set theory is relatively complex. Compared with the Bayesian theory, the D-S evidence theory has strong applicability and wide application. Therefore, urination and defecation were detected by the D-S evidence theory fusion method. 

Dempster proposed the upper and lower multi-valued mapping criteria to lay the foundation of D-S evidence theory [12], and then G. Shafer extended Dempster’s composition rule to more general cases through in-depth research, so that this theory can be applied to a wider range of fields in [13]. After conducting in-depth research on the D-S evidence theory proposed by G. Shafer, Chao Sun et al. proposed a MCDM weight method based on probability distribution negation, which reduces the uncertainty of human subjective factors [14]. Li proposed an improved D-S evidence theory based on orthogonal sum and standard deviation to solve the D-S evidence theory conflict problem, and verified its effectiveness [15]. Ma et al. proposed an improved evidence combination method by studying the combination principle of D-S evidence theory, and verified its fusion performance and reliability [16]. Based on the paradox problem of D-S evidence theory, Wang et al. proposed an improved classifier decision fusion method based on the D-S evidence theory [17]. Kisku D.R. et al. demonstrated the effectiveness of their new face recognition technology by integrating global and local matching methods through the D-S evidence theory [18].

The innovations of this paper are as follows: Aiming at the problem of the large working head and insufficient versatility of the existing excretion nursing equipment, the working head of the excretion nursing equipment designed in this paper adopts a flat design method, which can be used on an ordinary nursing bed. In order to improve the accuracy of urine and stool detection, temperature and humidity sensors and ammonia gas concentration sensors are used, and the D-S evidence theory algorithm is also used to improve the accuracy of urine and stool detection. The rest of this paper is organized as follows: Section 2 introduces the mechanical structure design of the excretion nursing equipment. Section 3 introduces the detection method based on the D-S evidence theory. Section 4 details an experimental study on the function of the excretion nursing equipment and the detection method of the urine and stool. Section 5 contains the conclusions.

## 2. Materials and Methods

This section will introduce the device we designed in detail. According to the design idea of the device, we will describe the mechanical system and control system in detail. The mechanical system mainly adopts the split design; the whole device is divided into the user directly wearing the working head and as the executive body of the host machine. The control system will introduce the hardware system and the software system: the hardware system mainly shows each module and the connection mode between modules, and the software system mainly introduces the detailed work flow.

### 2.1. Mechanical System Design

**Figure 2:**
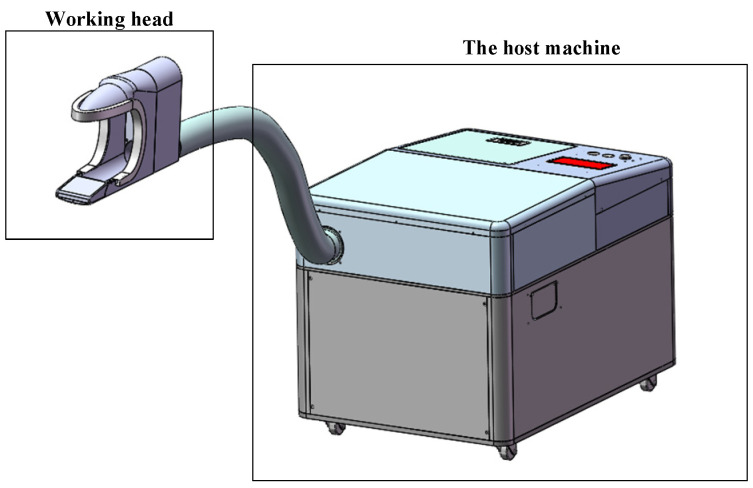
Mechanical structure of nursing equipment.

Our previous study revealed that user comfort and safety should be mainly considered when proposing a rehabilitation device [19]. The main target groups of the excretion nursing equipment include disabled and semi-disabled elderly people, users with limited mobility, elderly people who are bedridden for a long time, and people who are worried about infection after surgery. In order to meet the nursing needs of these people, the main functions of the excretion nursing equipment include: intelligent urine and stool detection function, urine and stool flushing function, human body washing function, drying function of human excretory sites, and overall deodorization of the urinal nursing equipment, disinfection function, and human–machine interaction function. The following will design the nursing equipment from the mechanical system and the control system according to the demand analysis. The overall structure of the excretion nursing equipment is shown in Figure 2, and its mechanical structure is mainly divided into two parts: the working head and the host. 

#### 2.1.1. Working Head of the Excretion Nursing Equipment

**Figure 3:**
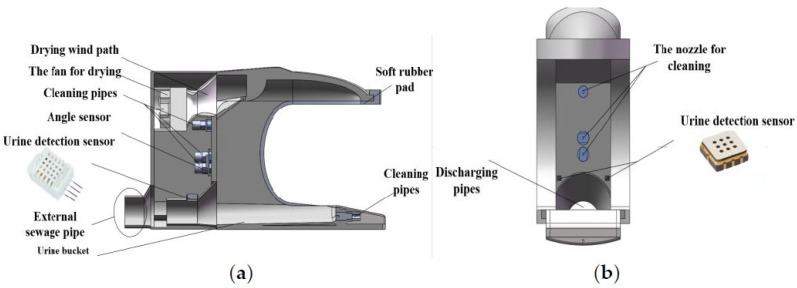
Mechanical structure of the working head. (**a**) Side view; (**b**) Front side view.

As shown in Figure 3, the working head includes a toilet bowl, urine and stool detection sensors, flushing water channels and nozzles, cleaning water channels and nozzles, drying air channels and nozzles, sewage pipes, and externally wrapped soft glue and other components. The working head toilet bowl is composed of the inner upper wall, rear wall, side wall, and bottom of the working head. The bottom is designed in a smooth arc shape, and maintains a 5° inclination angle with the horizontal plane, which is responsible for collecting the urine and stool discharged by the user. The urine and stool detection sensors use temperature, humidity sensors, and ammonia gas concentration sensors, which are respectively installed on the back wall of the toilet bowl and at the mouth of the sewage pipe. An angle sensor is installed behind the back wall of the toilet bowl to detect the side angle of the patient. The flushing water channel, the cleaning water channel and its nozzles are installed on the back wall of the bowl. The flushing nozzles are of two different types: conical and rectangular. The rectangular nozzles are used to flush small particles of stool and urine. The conical nozzles have high water pressure for breaking up hard-to-flush stools. The drying air duct is installed above the back wall of the toilet bucket, and a fan and a heater are also installed behind the air duct. The sewage pipeline is connected with the back wall of the toilet bowl, and is used to discharge the urine and stool collected by the toilet bowl into the dirt bucket. The soft rubber is wrapped on the convex groove on the outside of the toilet bowl to reduce the contact force between the human body and the hard shell of the working head. 

#### 2.1.2. Host Machine of the Excretion Nursing Equipment

**Figure 4:**
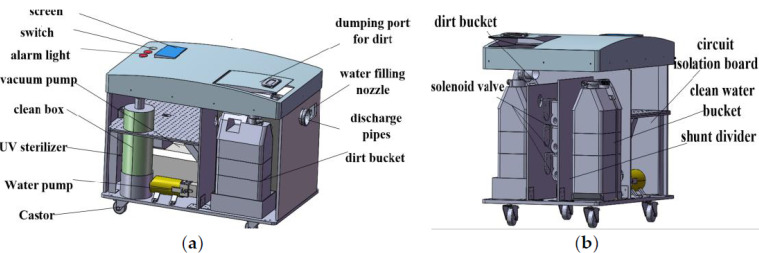
Mechanical structure of main engine. (**a**) Side view; (**b**) Front side view.

**Figure 5:**
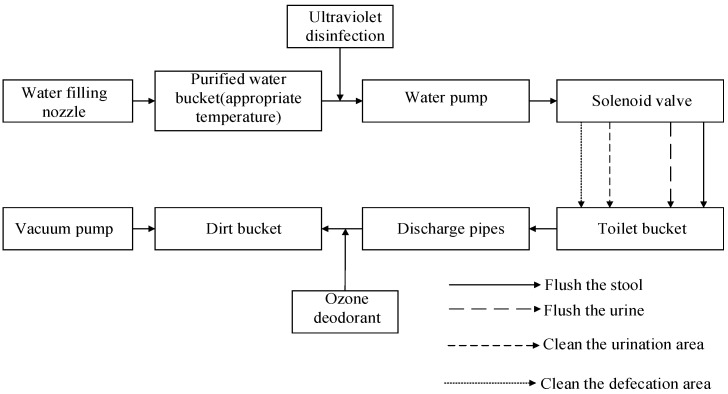
Component connection.

The host machine is the executive body of the excretion nursing equipment. The host machine is installed with the executive components and main controller of the nursing device, which is mainly responsible for the recovery of dirt, water purification, water purification, heating, negative pressure suction, negative ion deodorization, central control, and other main functions. The internal structure of the host machine is shown in Figure 4, including water purification pumps, ultraviolet sterilizers, vacuum pumps, water purification buckets, sewage buckets, cleaning boxes, solenoid valves, and other components. The water purification pump is installed at the bottom of the host machine, and is connected with the water purification bucket and the ultraviolet sterilizer through the water pipe, and the purified water is pumped out from the purification bucket and sent to the ultraviolet sterilizer for disinfection. The ultraviolet sterilizer is installed on the inner rear wall of the host, and is connected to the water purification pump and the pipeline nozzle through the water pipe. The vacuum pump and the dirt bucket are installed on the side of the clean water pump. The dirt bucket is connected to the vacuum pump, the cleaning box, and the sewage pipeline through the conduit. The dirt bucket stores the collected urine and dirty water. The vacuum pump is responsible for pumping negative pressure in the dirt bucket, and sucking urine and stool from the working head toilet into the dirt bucket through the sewage pipeline. The cleaning box is installed on the bottom side of the host machine, and the inside of the box is equipped with deodorizing activated carbon. The activated carbon is connected to the pipeline of the dirt bucket and is responsible for absorbing odor. The four-way solenoid valve is installed on the clapboard and is responsible for the on-off control of the water circuit. It is divided into four channels, namely the flushing the urine water channel, flushing the stool water channel, cleaning the urine excretion part water channel, and cleaning the stool excretion part water channel. The connection of components is shown in Figure 5. When performing different functions, open the corresponding solenoid valve to work. In order to avoid noise pollution caused by the operation of the internal components of the host, a layer of sound insulation sponge is laid in the shell of the host machine to absorb noise and reduce the impact of noise on the external environment.

**Figure 6:**
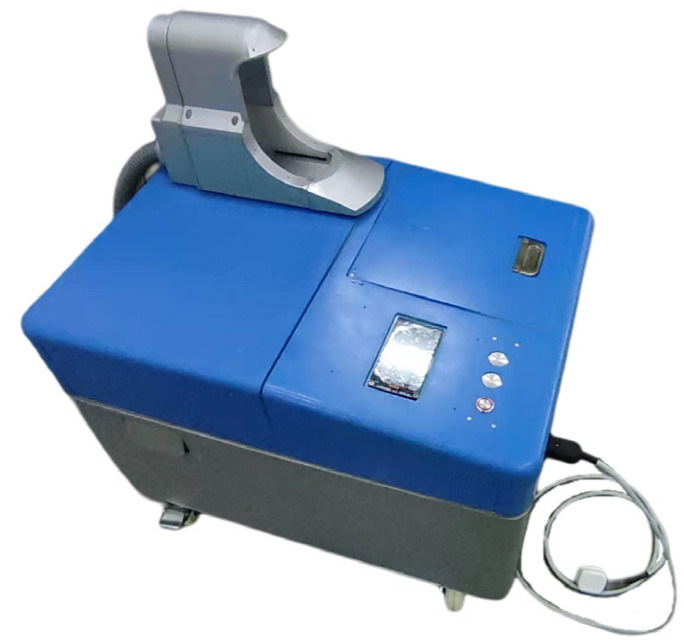
Prototype of excretion nursing equipment.

Through the design of the mechanical structure of the excretion nursing equipment in this section, an experimental prototype has been made. Figure 6 is a physical diagram of the prototype. 

### 2.2. Control System Design

The control system design is divided into two parts: hardware design and software design. The hardware design of the control system is analyzed from the aspects of component selection and function realization. The software design is analyzed from the operation process and function realization of the whole equipment. 

#### 2.2.1. Control System Hardware Design

The hardware of the excretion nursing equipment control system is mainly divided into the MCU central control module, intelligent detection module, flushing and cleaning control module, drying control module, and other parts. 

The MCU central control module uses the Stm32F4 chip as the main control core. Stm32F4 has a powerful peripheral interface, and the chip’s operating frequency can reach up to 168 Mhz, which can process the collected data information at a high speed, which satisfies the fast information collection and processing requirements of the experiments in this paper.

The intelligent detection module uses the temperature and humidity sensor and the ammonia gas concentration sensor as the main detection units. After the temperature and humidity sensor and the ammonia gas concentration sensor detect the change data, they are sent to the main control MCU to identify urine and stool by algorithms. The temperature and humidity sensor adopts DHT22. The humidity range that can be detected is 0–99.9%RH, the accuracy is ±2%RH, and the temperature range that can be detected is −40 °C–80 °C. The accuracy is ±0.5% °C. The ammonia gas sensor adopts the MEMS series GM-802B ammonia gas sensor, which can detect the concentration range from 1 ppm to 300 ppm (NH3). These sensor combinations can detect changes in temperature, humidity, and ammonia concentration caused by the user’s urination and defecation. The angle sensor is used to detect the patient’s sideways angle θ. The MPU6050 angle sensor is selected, and its detection angle range is ±180° for the X and Z axes, and ±90° for the Y axis. When the patient’s side angle is not in the range of −30°–30°, the sensor will send a detection signal to the main controller of the host, and the main controller will give an alarm to ensure the normal operation of the excretion nursing device.

The flushing and cleaning control module uses a 24 V diaphragm pump to extract clean water from the clean water bucket for flushing, cleans the waterway, and uses an ultraviolet sterilizer with an efficiency of 0.2 T/H to sterilize the clean water. At the same time, the module sends a level signal through the MCU to control the 24 V normally closed solenoid valve is switched on and off, and then the flushing and cleaning water circuits are controlled on and off, and the vacuum pump with a vacuum degree of 11,000 pa is used to pump the dirt into the dirt bucket. The water temperature in the clean water bucket is controlled by the thermostat and the heating rod to control the temperature in the bucket to the set appropriate temperature. Capacitive non-contact liquid level sensors are installed in the clean water bucket and the dirt bucket to detect the liquid level. When it is detected that the liquid level of the clean water tank is lower than the threshold or the liquid level of the dirt tank is higher than the threshold, it will send an electrical signal to the MCU. After the MCU receives the corresponding sensor signal, it will control the corresponding buzzer to alarm, reminding the nursing staff that they need to add clean water or replace the sewage bucket.

**Figure 7:**
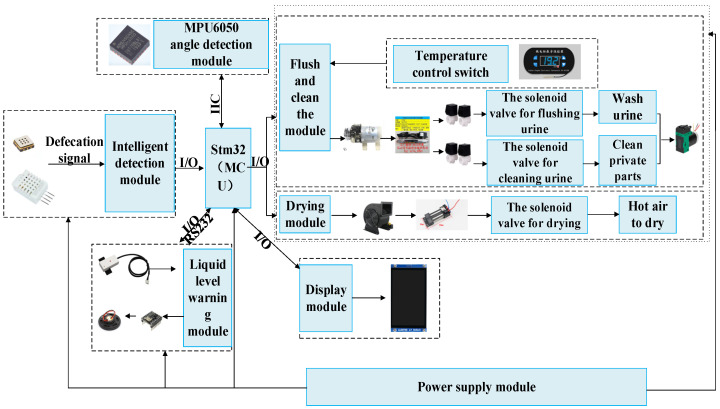
Control system structure diagram of excretion nursing equipment.

The drying control module uses a 24 V, 300 W PTC ceramic heating element to heat the air, and a 13,500 r/min motor as the drying fan. The MCU sends a level signal to control the on-off of the relay, so as to control the heating element and the motor. The human body is dried in the toilet bowl of the working head. The connection diagram between each module is shown in Figure 7. 

#### 2.2.2. Control System Software Design

**Figure 8:**
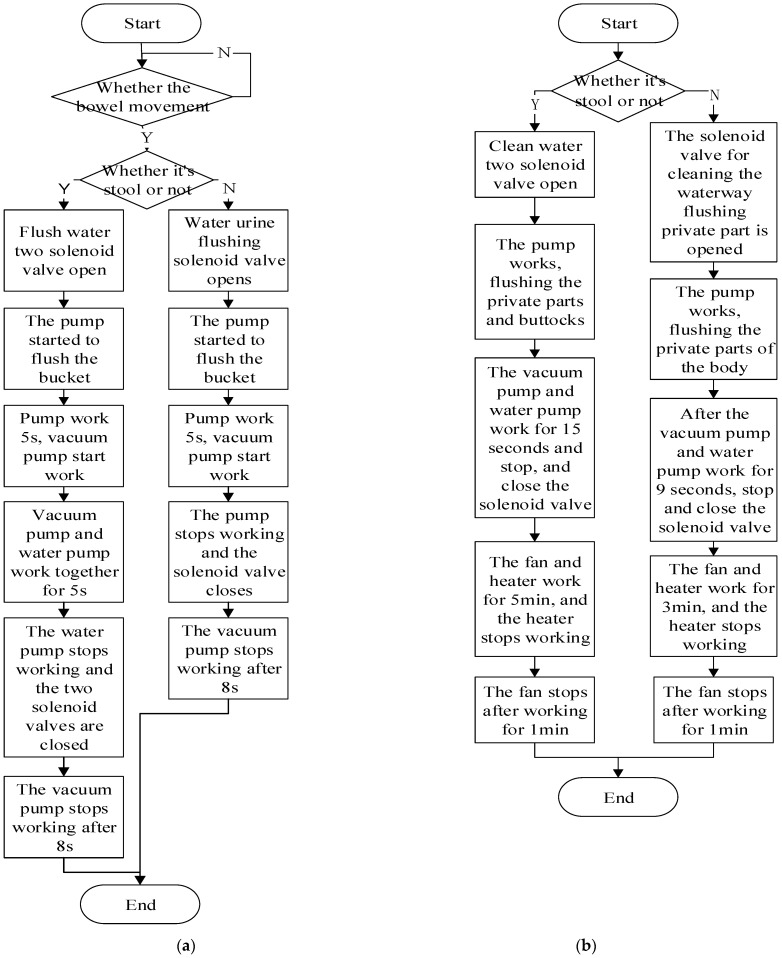
Flowchart of control system of excretion nursing equipment. (**a**) Test flush process; (**b**) Cleaning and drying process.

After the excretion nursing equipment is powered on, the central control module receives the data detected by the temperature, humidity, and ammonia gas concentration sensors, and then judges the type of the patient’s urination and defecation. After identifying the excretion type, the MCU will send an instruction to start the flushing process, and the urination and defecation are rushed from the toilet bowl to the dirt bucket. The detailed workflow is shown in Figure 8a. When the identification signal shows that the patient is expelling stool, the MCU will send a flushing command, which will open the solenoid valves for flushing the stool water circuit and the urine water circuit, and control the water pump to flush the inner toilet bowl of the working head (the flushing stool water circuit is used to flush large particles of dirt, and the flushing urine water circuit is used to flush the broken particle dirt). After the pump works for 5 s, the vacuum pump is controlled to start working at the same time, and the dirt and sewage on the working head are sucked into the dirt bucket by negative pressure. After the vacuum pump and the water pump work together for 5 s, the water pump stops working and closes the solenoid valves of the two water circuits. The vacuum pump continues to work for 8 s and then stops. When the identification signal shows that the patient excreted urine, a time delay of 10 s is performed to further detect and determine whether the patient excretes stool. After the patient defecates, the MCU sends a flushing command, opens the solenoid valve for flushing the urine water circuit, and controls the water pump to start flushing the bowl for 5 s. After that, the vacuum pump is controlled to work for 10 s to suck away the remaining sewage in the toilet bowl. Compared with flushing urine, the water pressure to flush the stool will be larger, which is convenient for breaking the stool, making flushing more convenient and further improving the cleanliness of flushing. The working time of the water pump and vacuum pump in the entire detection and flushing process are obtained by multiple experiments. 

After finishing the rinsing work, the excretion nursing equipment starts the cleaning and drying process, that is, the process of cleaning the excretory site with heated clean water and drying it with hot air. The operation process is shown in Figure 7b. The washing and drying process is also designed with two different processing modes for stool and urine. When the equipment detects that the patient has discharged the feces, it will wait for the washing to end, then open the solenoid valves for cleaning the stool and cleaning the urine, respectively, start the water pump to flush the patient’s urine and stool discharge port, and start the vacuum pump to suck the cleaned sewage into the sewage bucket. After the equipment works for 15 s, the solenoid valve and vacuum pump of the two cleaning water circuits is stopped and closed. After cleaning, the controller controls the fan and the heater to start working. The fan blows through the heater and sends the hot air into the working head. After 8 min, the heater stops working, and the fan stops working after 2 min, after which the drying process ends. When the equipment detects that the patient has discharged urine, the solenoid valve of the cleaning water channel for flushing the urine discharge site of human will be opened after the flushing process. The drying process for stool and urine works the same way.

Although the process of excretion nursing is complicated, for the healthcare professional, it is only necessary to ensure that the user has finished, and then operate the button on the host machine. The working condition information of the device can be displayed to the healthcare professional by the display screen. The healthcare professional only needs to check whether the equipment is working normally. If there are abnormal conditions, such as low water level in the bucket, the abnormal conditions can be removed according to the operation manual.

### 2.3. Automatic Detection of Urine and Stool Based on Multi-Sensor Fusion

Due to the performance of the sensor itself and the influence of the environment, there will be a certain deviation in the data information obtained by the sensor. When errors occur, the target cannot be identified effectively to obtain results [20]. Therefore, when the sensor is used for direct detection in the nursing device, it is unable to accurately detect whether the user excretes stool or urine. In order to solve this problem, this paper puts the detection results of the temperature and humidity sensor and the ammonia gas sensor into the D-S evidence theoretical model for fusion processing to obtain the final detection result, so as to improve the detection accuracy. As a multi-information source fusion rule, this algorithm can process multiple independent information to obtain the confidence of the proposition, and obtain the accuracy or uncertainty of the final proposition result through comprehensive analysis of the confidence [21,22].

**Figure 9:**
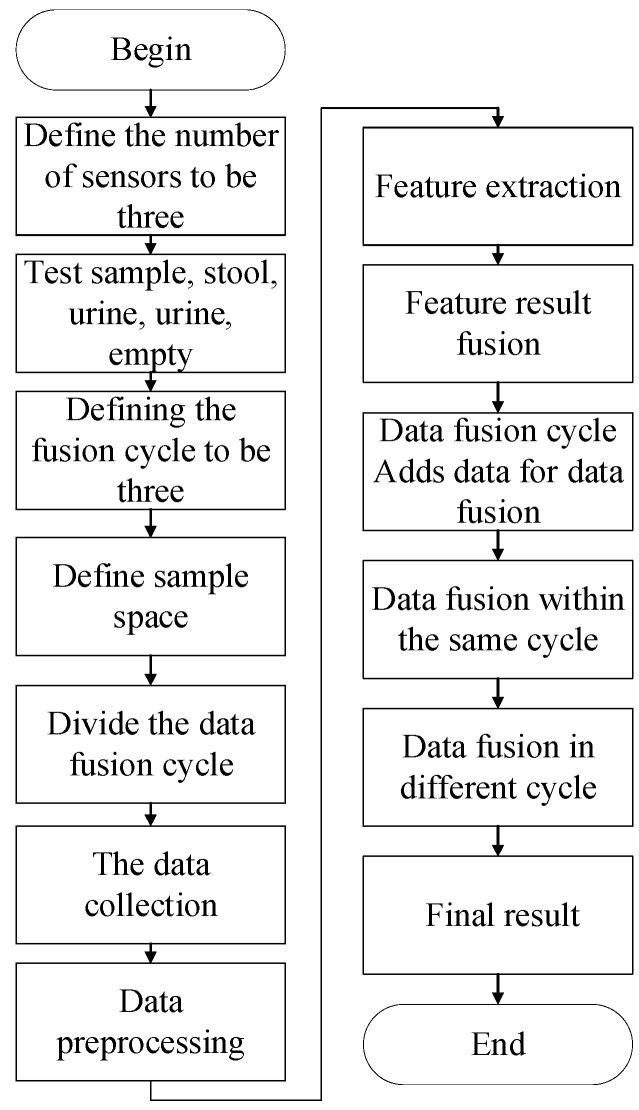
D-S evidence theory model running flowchart.

The D-S evidence theory fusion steps are mainly divided into four steps: sensor data collection, data feature extraction, data fusion, and result confirmation [23]. The specific flowchart of the application of D-S evidence theory in this paper is shown in Figure 9.

As mentioned above, this paper uses three kinds of sensors for detection, which defines three types of information source subjects in the D-S evidence theory model. According to the general experience of D-S evidence theory, in order to make the fusion result more favorable for analysis, it is often necessary to perform multiple fusions, which is called multiple fusion cycles. Multiple fusion cycles do not change the results, but only polarize the probability assignment and make the results more salient. In this study, there are three fusion cycles according to the above-mentioned experience, that is, when each sensor collects data for detection, it collects data at nearby moments twice, so that three sets of complete experimental data are finally obtained. Since there are only four final detection results, namely urine (event A), stool (event B), urine and stool (event C), and no detection (event D), the sample space can be determined. Next, multiple sensors collect data, and integrate these data for preprocessing. During the collection, the average value of multiple groups of data is taken, and the obviously unreasonable data are eliminated. The next step is to perform feature extraction on the sorted data, and extract the representative information in the data that can characterize the results. Then, using the data fusion rule of the D-S evidence theory, each cycle is first fused, and finally, the three sets of fusion results are fused, after which the target result is obtained.

According to the above process description, in order to use the D-S evidence theory fusion rule for modeling, it is necessary to preprocess the data obtained by three sensors. First, a large amount of data from three sensors for four possible situations are collected, and four sample spaces are constructed using the data; the data range of the four situations are obtained through analysis as the basis for judging the results (the specific operations are shown in the experiment section). Next, the ratio of the experimental data obtained by each sensor in the actual detection and the judgment basis of the result is taken as the probability of the occurrence of the corresponding event. From this, the basic probability distribution function of urine (event A), stool (event B), urine and stool (event C), and no detection (event D) detected by the temperature sensor is $M_{1}(A)$, $M_{1}(B)$, $M_{1}(C)$, and $M_{1}(D)$, respectively. The basic probability distribution functions of the humidity and ammonia concentration sensors are $M_{2}(A)$, $M_{2}(B)$, $M_{2}(C)$, and $M_{2}(D)$, and $M_{3}(A)$, $M_{3}(B)$, $M_{3}(C)$, and $M_{3}(D)$, respectively.

Then, the D-S evidence theory data fusion formula is used to fuse the characteristic result data. The composite result of two subjects for event A is equal to the ratio of the sum of the product of the probability distribution function values of all events intersecting as A in the data sample space of the two subjects to the normalization coefficient. Taking the result data fusion of temperature sensor and humidity sensor for event A as an example, the synthesis formula is shown in Equation (1):

(1) $$\left(M_{1}⊕M_{2})(A)=\frac{1}{K}\sum_{A\cap C=A}M_{1}(A)*M_{2}(C\right)$$

The orthogonal symbol “⊕” on the left of the equal sign represents the data fusion between different subjects for an event in the sample space, which is the fusion result of the temperature and humidity sensors for urine (event A). The right side of the equal sign is the ratio of the sum of the product of the probability distribution function values of all events intersecting A to the normalized coefficient K. Similarly, the fusion formula (2) for event B is:

(2) $$\left(M_{1}⊕M_{2})(B)=\frac{1}{K}\sum_{Β\cap C=B}M_{1}(B)*M_{2}(C\right)$$

In the above two equations, K is the normalization coefficient. Taking the result data fusion of temperature sensor and humidity sensor for event a as an example, the calculation method of K is shown in formula (3):

(3) $$K=1-\sum_{A\cap C=\emptyset }M_{1}(A)*M_{2}(C)=\sum_{A\cap C\neq \emptyset }M_{1}(A)*M_{2}(C)$$

The normalized coefficient K can be understood as the sum of the product of all probability distribution function values whose intersection is not a null set in the sample space of the temperature and humidity sensors. In this study, it is the sum of $M_{1}(A)$ ∗ $M_{2}(A)$, $M_{1}(A)$ ∗ $M_{2}(C)$, $M_{1}(B)$ ∗ $M_{2}(B)$, $M_{1}(C)$ ∗ $M_{2}(C)$, $M_{1}(C)$ ∗ $M_{2}(B)$, $M_{1}(C)$ ∗ $M_{2}(C)$, and $M_{1}(D)$ ∗ $M_{2}(D)$.

After the fusion is completed according to formula (1), the fusion result in one cycle can be obtained by fusing the result with the ammonia sensor, that is, a probability matrix of four events. Then, repeat the above steps until the fusion result in three cycles is obtained. Finally, the three results are fused to the final probability matrix, in which the event with the highest probability is the result event.

## 3. Results

In order to fully verify the feasibility of our equipment, the experiment will be divided into two parts: a detection experiment and a functional experiment. The former is mainly to verify whether the D-S evidence theory can improve the accuracy of stool and urine detection, and the latter is mainly to verify whether the function of our device can be realized.

### 3.1. Detection Experiments

**Figure 10:**
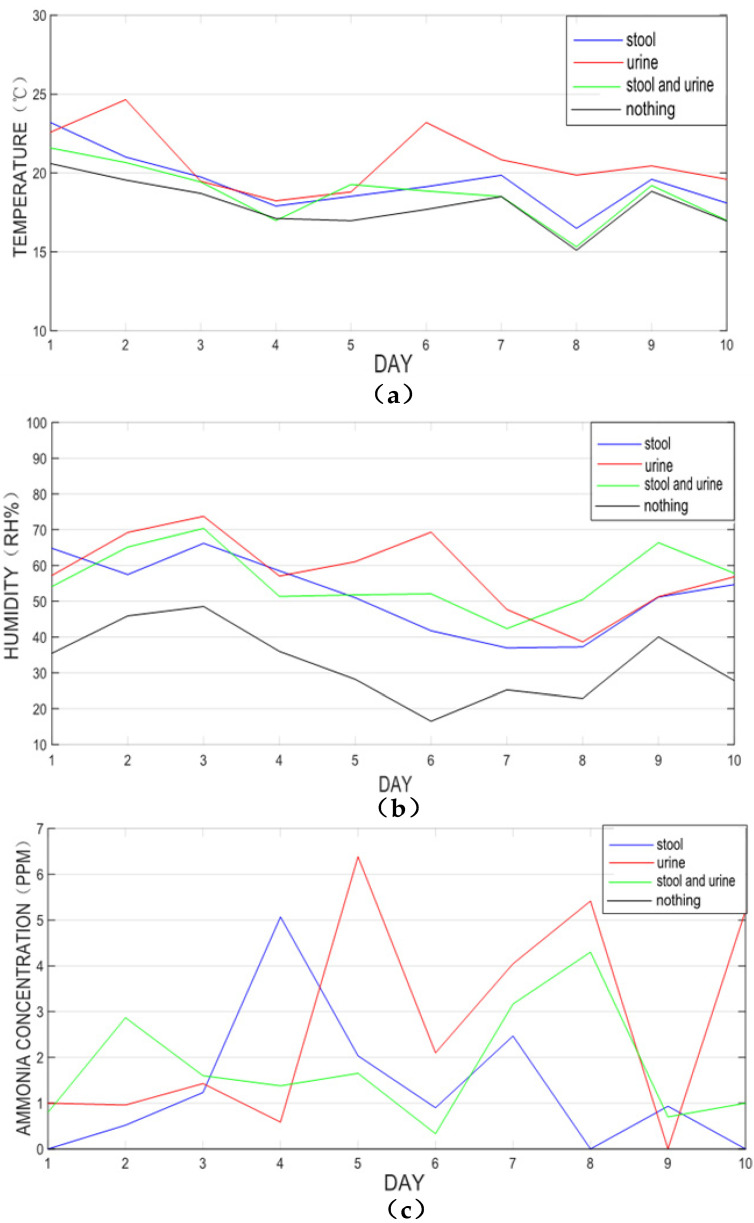
The data value of sample space. (**a**) Temperature data of four sample spaces. (**b**) Humidity data of four sample spaces; (**c**) Ammonia concentration data of four sample spaces.

In order to obtain the judgment basis of the results, firstly, the sensor is used to detect and identify the user’s urine and stool directly. In this paper, a 10-day experiment was conducted. According to the four experimental results, a total of 800 datapoints from three sensors were collected and divided into four groups (urine group, stool group, urine and stool group, and no detection group) to establish a sample space. Since the data will be collected multiple times in a short period of time, the data with significant fluctuations are excluded and the average value is taken as one data; all data have been processed similarly. Next, the multi-group averages of the target samples in the data space are used for analysis [24], and the humidity data of all samples in the four sample spaces for 10 days are obtained, as shown in Figure 10a. Figure 10b displays the temperature data. Figure 10c is all the ammonia concentration data in the four sample spaces for 10 days (under normal circumstances, the ammonia concentration is 0, which coincides with the *x*-axis).

Through the data collected from the detection target, the value range of the target urine and stool is determined. According to the data obtained from multiple sets of experimental data, it can be seen that the temperature and humidity values detected in the urine are relatively high, and the ammonia concentration in the stool is maintained at around 1 ppm. The humidity of stool and urine is between urine only and stool only, and the ammonia concentration in the blank condition is 0. Based on this, a set of measurement standards is formulated as the detection basis for identifying the occurrence of urination and defecation, as shown in Table 1 (with the indoor temperature and humidity as the reference point, set temperature as x°, humidity as y RH%, and ammonia concentration as z PPM). The temperature range of stool is 0.5°–2° higher than room temperature, the range of urine is more than 1.8° higher than room temperature, and the range of stool and urine is 0°–1°. The test result with a temperature value of 0°–1° higher than room temperature is defined as urine and stool. If the humidity value is 5%–12% higher than the indoor humidity, the test result is defined as stool, the humidity value is higher than 20% above the indoor humidity, the test result is defined as urine, and the humidity value is 12%–20% higher than the indoor humidity, while the test result is defined as urine and stool. When the ammonia concentration value is 1 ppm–2 ppm higher than the ammonia concentration in the air, the test result may be stool or stool and urine. The ammonia concentration value is more than 2 ppm higher than the ammonia concentration in the air, and the test result is defined as urine.

Ten groups of detection data are selected randomly from three sensors and put into the sample space, and the urine and stool are identified directly using the sensor data. Experimental results show that the probability of correctly identifying the urine and urine is 70.83%, and there is a situation that the urine and stool are not clearly identified, which will make the nursing equipment have problems in the subsequent washing, cleaning, and drying operations. After the pretreatment of 10 groups of experimental data as described above, the D-S evidence theory is introduced. According to the data fusion process of the D-S evidence theory in Section 2, data fusion decisions are made for temperature, humidity, and ammonia concentration data. 

According to the results obtained in Table 2, using the statistical method of Paired χ^2^ Test for verification. The χ^2^ was calculated and *p* < 0.05 was obtained according to the χ^2^ value, which proved that the results were statistically significant. At the level of α = 0.05, it was considered that the accuracy of the two methods was different, and the accuracy of the method adopted in this paper reached 90%, which was significantly higher than that of the traditional method. 

### 3.2. Function Test

Experiments were also performed to test the overall function of the excretion nursing equipment. The results show that the nursing equipment can achieve the functions of flushing the dirt in the toilet bowl and washing and drying the human body, and it can satisfy the needs of helping patients to achieve self-care. At the same time, the designed excretion nursing equipment also realizes the function of detecting the patient’s sideways angle and reminding the patient to restore their posture. The liquid level of the water bucket and the dirt bucket can be detected, and when the liquid level of the clean water bucket is lower than the threshold or the liquid level of the dirt bucket is higher than the threshold, the alarm reminder function is performed.

### 3.3. Safety Instructions

As a device to be introduced in the health field, safety issues cannot be ignored. According to the provisions of anti-electric shock and other dangerous situations in Part I: General Safety Requirements of “Medical Electrical Equipment”, the accessible parts of our equipment are insulating materials. In the host part, according to “Shell Protection Level (IP Code)”, the host machine shell of our device has reached IP44 level, which has good protection level for solid liquid. As the working head is directly in contact with human skin, we choose medical soft glue as the material to wrap on the raised groove outside the toilet bowl, according to the biocompatibility requirement of the “Biological Evaluation of Medical Devices”, which states that the skin irritation should not be higher than very slight irritation, so as to improve the use experience as much as possible without causing damage to the user’s skin.

## 4. Discussion

Many scholars have done a lot of theoretical research on the problem of excretion nursing, but lack of device to apply these theories. At present, the relatively mature products include the Smilet sleeping automatic excretion treatment robot developed in Japan (Figure 1a) and the product of the Illinois nursing robot in Suzhou, China (Figure 1b). These two types of equipment are also of split design, and are structurally divided into two parts: working head and host machine. It also has the functions of detection, washing, cleaning, and drying. However, they used a single sensor detection method, which directly based on the amount of ammonia as the basis for determining urine and stool, and had a low success rate. In terms of the setting of the working process, the two types of devices are not equipped with the liquid level alarm system of the water purification bucket and the dirt bucket, but rely on manual inspection, which may cause inconvenience in the use of the equipment.

From the experimental results, the design of this paper has achieved a good effect, but this study still has some shortcomings: first, there are few subjects, and there might be significant differences in the data related to urine and stool of different subjects, which makes the test results not general, and it is impossible to identify the urine and stool of most users accurately. Not only that, the subjects’ daily different diets will also have an impact on urine and stool, which will further cause confusion. In the later stage of the experiment, it is also necessary to improve the detection equipment and classify and recognize the errors. At the same time, it is necessary to expand the sample size to establish a large database, and neural network technology can be used for integrated analysis to improve the recognition accuracy. In terms of function, the device still has room to be optimized for incontinent users, because wearing the device for a long time will affect the skin of patient’s private parts; thus, the nursing staff should be mindful to temporarily remove the device after completing a cleaning or wearing it for a certain time.

As a matter of fact, the D-S evidence theory we used can also be applied to other medical problems, such as medical diagnosis. In the future, we will continue to upgrade the equipment and expand the scope of applications, such as home daily care and so on.

## 5. Conclusions

This study designed an excretion nursing equipment that uses temperature, humidity, an ammonia concentration sensor, and the D-S evidence theory to detect and identify patients’ urine and stool. It has the function of detecting and identifying patients’ urine and stool, washing, cleaning, and drying the user’s skin, and can help the disabled and semi-disabled elderly, as well as bedridden patients with poor mobility to realize self-care of urine and stool. Based on the detection and function experiments results, the urine and stool recognition and detection algorithm based on the D-S evidence theory used in this paper can obtain a better recognition accuracy than the traditional detection methods, and the excretion nursing equipment can realize the function of excretion care for patients.

## Figures and Tables

**Table 1:** Basis for testing urine and stool.

	Stool	Urine	Stool and Urine	No Detection
Temperature (°C)	0.5 < x < 2	1.8 < x	0 < x < 1	0
Humidity (RH%)	5 < y < 12	20 < y	12 < y < 20	0
Ammonia concentration (ppm)	1 < z < 2	z > 2	1 < z < 2	0

**Table 2:** D-S evidence theory data fusion table.

Num	Humidity (rh%)	Temperature (°C)	Ammonia Concentration (ppm)	Result
1	65.9	20.6	3.0	[Stool and urine]
2	80.4	19.7	1.0	[Stool]
3	63.7	21.1	0.0	[Stool]
4	67.0	17.9	7.0	[urine] (error)
5	55.4	19.2	2.0	[urine]
6	76.0	23.4	2.0	[urine]
7	42.0	19.7	3.0	[Stool]
8	43.3	19.8	8.0	[urine]
9	22.9	15.1	0.0	[no detection]
10	52.8	19.6	5.0	[urine]

## Data Availability

The data presented in this study are available on request from the corresponding author.
